# The economic value of changing mortality risk in low- and middle-income countries: a systematic breakdown by cause of death

**DOI:** 10.1186/s12916-021-02029-x

**Published:** 2021-07-16

**Authors:** Aayush Khadka, Stéphane Verguet

**Affiliations:** Department of Global Health and Population, Harvard T. H. Chan School of Public Health, Boston, MA 02115 USA

**Keywords:** Economic burden of disease, Mortality reduction, Non-communicable diseases, Communicable diseases, Priority setting, Low- and middle-income countries

## Abstract

**Background:**

We develop a framework for quantifying monetary values associated with changes in disease-specific mortality risk in low- and middle-income countries to help quantify trade-offs involved in investing in mortality reduction due to one disease versus another.

**Methods:**

We monetized the changes in mortality risk for communicable and non-communicable diseases (CD and NCD, respectively) between 2017 and 2030 for low-income, lower-middle-income, and upper-middle-income countries (LICs, LMICs, and UMICs, respectively). We modeled three mortality trajectories (“base-case”, “high-performance”, and “low-performance”) using Global Burden of Disease study forecasts and estimated disease-specific mortality risk changes relative to the base-case. We assigned monetary values to changes in mortality risk using value of a statistical life (VSL) methods and conducted multiple sensitivity analyses.

**Results:**

In terms of NCDs, the absolute monetary value associated with changing mortality risk was highest for cardiovascular diseases in older age groups. For example, being on the low-performance trajectory relative to the base-case in 2030 was valued at $9100 (95% uncertainty range $6800; $11,400), $28,300 ($24,200; $32,400), and $30,300 ($27,200; $33,300) for females aged 70–74 years in LICs, LMICs, and UMICs, respectively. Changing the mortality rate from the base-case to the high-performance trajectory was associated with high monetary value for CDs as well, especially among younger age groups. Estimates were sensitive to assumptions made in calculating VSL.

**Conclusions:**

Our framework provides a priority setting paradigm to best allocate investments toward the health sector and enables intersectoral comparisons of returns on investments from health interventions.

**Supplementary Information:**

The online version contains supplementary material available at 10.1186/s12916-021-02029-x.

## Background

Emerging economies and low- and middle-income countries face the concurrent challenge of high premature mortality due to communicable diseases (CDs) as well as non-communicable diseases (NCDs) [1–5]. Since they face this double burden of disease and since all developing countries face severe resource constraints, understanding the evolution of these diseases in the short-to-medium term and the trade-offs involved in reducing the burden of one disease versus another is critical for policymakers.

In this vein, several academic and non-academic institutions have produced disease- and country-specific mortality and life expectancy forecasts [6–11]. From a policymaking standpoint, translating changes in health outcomes into currency-denominated units may be useful because, first, it allows the comparison of changes in life expectancy/mortality against policy/intervention costs, which can yield return on investment (ROI) figures that can be used to compare policies/interventions across sectors; second, policymakers can combine such monetary estimates with information on changes in other welfare outcomes and across sectors beyond the health sector (e.g., education, agriculture) since they are often estimated in monetary terms as well; and, third, it can facilitate the calculation of a country’s full income (usually defined as the sum of a country’s gross domestic product and value of changes in mortality), which can be particularly useful for health policymakers to motivate greater investments in improving societal health [12–14].

Along these lines, Jamison and colleagues wrote a seminal paper monetizing the economic benefits of reducing infectious, child, and maternal mortality rates between 2015 and 2030 and demonstrated high ROI (in the order of around 9–20 to 1) from financing key targeted interventions addressing these diseases [14]. In this paper, we advance this work by developing an analytic framework that quantifies, in monetary terms, changes in annual age-sex-specific mortality due to various NCDs and CDs [15]. We label our monetary estimates income-equivalent monetary value (IEMV) to clarify the fact that the monetary estimates produced in this paper are assigned to a non-tradable good that is health. We focus on presenting our methodology for estimating the IEMV and illustrate its application by using publicly available estimates on mortality rates, national income, and population across low-income countries (LICs), lower-middle-income countries (LMICs), and upper-middle-income countries (UMICs) between 2017 and 2030, the end date of the Sustainable Development Goals (SDGs).

## Methods

Our approach for estimating disease-specific IEMV involved three steps. First, we contrasted mortality risk under a high- and low-performance mortality trajectory with a reference trajectory for all countries (LICs, LMICs, and UMICs). Second, we used value of a statistical life (VSL) methods recommended for LICs, LMICs, and UMICs to assign monetary values to these changes in mortality risk. Third, we conducted multiple sensitivity analyses to assess the robustness of our estimates to assumptions made when estimating VSL.

LICs, LMICs, and UMICs were defined based on the 2016 World Bank country income group classification [16]. Specifically, LICs, LMICs, and UMICs were respectively defined as countries with gross national income (GNI) per capita (in 2016 USD) lower than $1005, between $1006 and $3995, and between $3996 and $12,235. We applied our approach to eight disease categories in these countries as classified by the Global Burden of Disease (GBD) study: (1) neoplasms; (2) cardiovascular diseases (CVDs); (3) chronic respiratory diseases; (4) diabetes, blood, urogenital, and other endocrine diseases; (5) mental disorders; (6) injuries; (7) CDs; and (8) maternal, neonatal, and nutritional diseases (see Additional file 1: Web Appendix Table A1).

The following sub-sections provide a summary description of each of the three steps involved in estimating the IEMV. We provide more details regarding each step with all the equations used in Additional file 1: Web Appendix Detailed Methods.

### Step 1: Estimating mortality risk changes between high/low-performance trajectories and the base-case trajectory

We downloaded mortality rate estimates associated with the GBD 2016 study from the GBD Foresight web application (URL: https://vizhub.healthdata.org/gbd-foresight/) to define the reference, low-, and high-performing mortality trajectories [17]. In accordance with GBD exercises, we defined the age-sex-specific mortality rate for a given disease *d* in country *c* in year *y* as the number of deaths due to the disease divided by the population in age interval [*a*; *a* + *n*), where *n* = 5 (e.g., 0–4 years; 5–9 years) except in the case of the final age group which was an open interval for individuals 95 years and older.

We defined three mortality trajectories for each of the eight disease categories in country *c* in year *y* for every age-sex-specific group between 2017 and 2030. The first trajectory (“base-case”) was the reference trajectory and referred to the annual mortality rate forecasts from the GBD 2016 study [17]. The second trajectory (“high-performance”) was defined as achieving, by 2030, the mortality rates that corresponded to the 90th percentile of the lowest mortality rates of countries in the next higher income group. The third trajectory (“low-performance”) was defined as achieving, by 2030, the rates that corresponded to the 10th percentile of the lowest mortality rates of countries in the next higher income group.^1^

We calculated age-sex-specific mortality rates under the high- and low-performance trajectories for each year between 2017 and 2029 by log transforming the GBD-provided mortality rate in 2016 and the target mortality rate in 2030 and then linearly interpolating between these two points. In doing so, we assumed that mortality rates across disease categories were independent of one another.^2^

After defining the high- and low-performance trajectories, we converted mortality rates into the risk of death conditional on surviving up to a given age group. To estimate this conditional probability of death for a given disease category, we made two assumptions: first, we assumed that when evaluating the high- or low-performance trajectory for disease category *d*, the mortality rates for the remaining seven disease categories remained the same as under the base-case. Second, we assumed that mortality rates across all eight diseases could be added to get the overall age-sex-specific mortality rate (Additional file 1: Equation A1, Web Appendix Detailed Methods). Given these assumptions, we estimated the total age-sex-specific mortality rate under the high- and low-performance trajectories for each disease and transformed it into the conditional probability of death using standard demographic methods (Additional file 1: Equation A2, Web Appendix Detailed Methods).

Finally, we estimated the difference in mortality risk between the high/low-performance trajectory and the base-case. Jamison and colleagues as well as the published VSL literature generally provide monetary values for changes in one standardized mortality unit (SMU). A SMU refers to a change in the risk of death of 1 per 10,000 (i.e., 10^−4^) [15, 18]. Accordingly, we re-scaled the difference in mortality risk in terms of SMUs (Additional file 1: Equation A3, Web Appendix Detailed Methods).

### Step 2: Estimating the IEMV of changes in mortality risk

The monetary value associated with a SMU is called the value of a standardized mortality unit (VSMU). For each disease category, the first step in estimating the age-sex-year-country-specific VSMU was to estimate the VSL for each country in our sample in each year over the study period. The VSL is a proxy for the willingness to pay for a small reduction in the risk of mortality (i.e., exchanging income against mortality risk reduction) [19].

Since VSL estimates are not readily available for all LICs, LMICs, and UMICs, we followed the VSL literature and estimated the country-year-specific VSL (denoted *VSL*_*c*, *y*_ for country *c* in year *y*) by scaling the VSL in the USA in 2015 (denoted *VSL*_*base*_) by the ratio of the gross national income (GNI) per capita in each country-year (denoted *I*_*y*, *c*_) and the US GNI per capita in the same year (denoted *I*_*y*, *base*_) [19]. That is:

1 $$ VS{L}_{c,y}= VS{L}_{base}\times {\left(\frac{I_{y,c}}{I_{y, base}}\right)}^{\varepsilon}\kern0.5em $$

In Eq. 1, *ε* refers to the VSL income elasticity, that is the percentage change in VSL for a percentage change in income [20]. Since the GNI per capita ratio increases, on average, with country income group, Eq. 1 implies that LICs will have lower VSL estimates than LMICs, and LMICs than UMICs [19, 20].

GNI data were available from the World Bank’s World Development Indicators database until only 2017 [21]. Thus, we estimated the country-year-specific GNI per capita for years between 2018 and 2030 by following a three-step process. First, using data from 2010 to 2017, we computed the exponential growth rate in GNI per capita for each country in our sample. Next, we derived the mean growth rate in the years before 2017 within each country income group (within LICs, LMICs, and UMICs, respectively). Third, we used the growth rate formula and the derived country income group-specific mean growth rate to estimate the yearly GNI per capita for each country between 2018 and 2030.^3^

We plugged in the estimated GNI per capita into Eq. 1 to estimate country-year-specific VSL. Then, using this VSL estimate, we estimated the VSMU at age group [35, 40) in the same country-year (Additional file 1: Equation A5, Web Appendix Detailed Methods). We drew from Jamison et al. and used age group [35, 40) as the reference age category from which to determine the VSMU at all other age groups [15]. We then estimated the VSMU for all age groups in each country-year by scaling the VSMU for age group [35, 40) by the ratio of each age group’s estimated life expectancy and the life expectancy for age group [35, 40). That is,

2 $$ VSM{U}_{c,y}(a)=\frac{e_{a,s,y,c}}{e_{35,s,y,c}} VSM{U}_{c,y}(35)\kern0.5em $$

where *e*_*a*, *s*, *y*, *c*_ refers to the age-sex-year-country-specific life expectancy and *VSMU*_*c*, *y*_(*a*) refers to the VSMU at age group [*a*; *a* + 5). We estimated life expectancy for all age-sex groups in each country-year by converting the GBD mortality rate projections under the base-case into age-sex-specific survival probabilities and then constructing life tables using these probabilities. Equation 2 mechanically leads to deaths in younger age groups being valued more than deaths in older age groups.

All VSMU estimates were expressed in 2015 US dollars and were undiscounted. We multiplied the change in mortality risk between the high/low-performance trajectory and the base-case by the respective VSMU estimate to calculate the IEMV. When age-sex-year-country-specific mortality risks were lower relative to the base-case, the IEMV estimates were positive; when they were higher, the IEMV estimates were negative; when they were the same, the IEMV estimates equaled zero.

### Step 3: Sensitivity analyses

We followed best practices outlined by Robinson and colleagues and conducted three sensitivity analyses [19, 22, 23]. All three sensitivity analyses involved changing the GNI per capita ratio in Eq. 1. Specifically, the first scenario set *ε* = 1.5, *VSL*_*base*_ = $9.4 million, and US GNI per capita to $57,900. We set a ceiling to this ratio which we describe in Additional file 1: Web Appendix Detailed Methods. In the second and third scenarios, we set *ε* = 1 and fixed the GNI per capita ratio to 160 and 100, respectively.

### Aggregate level reporting of the income-equivalent monetary value

We summarized our findings by aggregating by age-group-year across all countries within an income group (LICs, LMICs, and UMICs). To do so, we estimated the age-group-weighted mean VSMU for each age-group-year. To compute the age weights, we used country-specific projections available from the United Nations Population Division’s World Population Prospects [9]. However, since age-group-specific population projections were only available for 2020, 2025, and 2030, we aggregated results and estimated 95% uncertainty ranges (URs) for these 3 years only.

All computational simulations were conducted using Stata MP/16.1 [24].

## Results

Table 1 lists the countries included in our study and their corresponding income group. Nine countries were not included due to a lack of GNI data, population data, or mortality rate estimates. Countries excluded from this analysis and the reasons for their exclusion are listed in Additional file 1: Web Appendix “Countries excluded from our analysis”.

**Table 1:** Countries included in the analysis categorized according to the World Bank income classification for 2016

Low-income countries		Lower-middle-income countries				Upper-middle-income countries			
*GNI per capita ≤ $1005*		*GNI per capita between $1006 and $3995*				*GNI per capita between $3996 and $12,235*			
Afghanistan	Madagascar	Angola	Honduras	Nicaragua	Vanuatu	Albania	Fiji	North Macedonia	Turkey
Benin	Malawi	Armenia	India	Nigeria	Vietnam	Argentina	Gabon	Montenegro	St. Vincent and the Grenadines
Burkina Faso	Mali	Bangladesh	Indonesia	Pakistan	West Bank and Gaza	Azerbaijan	Equatorial Guinea	Mauritius	Venezuela, RB
Burundi	Mozambique	Bhutan	Jordan	Papua New Guinea	Yemen	Bulgaria	Grenada	Malaysia	Samoa
Central African Republic	Nepal	Bolivia	Kenya	Philippines	Zambia	Bosnia and Herzegovina	Guyana	Namibia	South Africa
Chad	Niger	Cabo Verde	Kiribati	Sao Tome and Principe		Belarus	Croatia	Panama	Turkey
Comoros	Rwanda	Cambodia	Kyrgyzstan	Solomon Islands		Belize	Iran, Islamic Rep.	Peru	
Democratic Republic of the Congo	Senegal	Cameroon	Laos	Sri Lanka		Brazil	Iraq	Paraguay	
Eritrea	Sierra Leone	Congo, Rep.	Lesotho	Sudan		Botswana	Jamaica	Romania	
Ethiopia	South Sudan	Cote d'Ivoire	Mauritania	Swaziland		China	Kazakhstan	Russian Federation	
The Gambia	Tanzania	Egypt	Micronesia	Tajikistan		Colombia	Lebanon	Serbia	
Guinea	Togo	El Salvador	Moldova	Timor-Leste		Costa Rica	Libya	Suriname	
Guinea-Bissau	Uganda	Georgia	Mongolia	Tunisia		Dominican Republic	St. Lucia	Thailand	
Haiti	Zimbabwe	Ghana	Morocco	Ukraine		Algeria	Maldives	Turkmenistan	
Liberia		Guatemala	Myanmar	Uzbekistan		Ecuador	Mexico	Tonga	

Note: *GNI* gross national income

Table 2 shows age-sex-specific VSMU by country income group averaged over the study period (2017–2030). VSMU estimates presented can be interpreted as the average monetary value equivalent to a 10^−4^ change in the risk of death in each age-sex-country income group over the study period. For example, a 10^−4^ change in the risk of death among under-five females in LICs was valued (over the study period, on average) at $59 (95% UR $54–64), in comparison to $231 ($219–244) in LMICs and $552 ($532–572) in UMICs. Beyond the fact that they increased by age and country income group, our VSMU estimates were also slightly higher for males relative to females in the younger age groups in LMICs and UMICs. This reflected differences in the life expectancy ratio as defined in Eq. 2 between males and females. VSMU estimates across the three sensitivity analysis scenarios are provided in Additional file 1: Web Appendix Tables A2-4. Additional file 1: Web Appendix Table A5 presents the average VSL estimate by country income group over the study period.

**Table 2:** Age-sex-specific value of standardized mortality units (VSMU) averaged across the study period and stratified by World Bank country income group (2015 USD)

	Low-income country		Lower-middle-income country		Upper-middle-income country	
Age group	Females	Males	Females	Males	Females	Males
< 5 years	59 (54–64)	59 (54–64)	231 (219–244)	239 (226–252)	552 (532–572)	575 (554–596)
5–9 years	55 (50–59)	55 (51–60)	218 (206–230)	224 (212–236)	518 (499–536)	536 (517–555)
10–14 years	51 (47–55)	51 (47–55)	204 (193–215)	208 (197–220)	483 (465–500)	496 (479–514)
15–19 years	48 (44–51)	48 (44–51)	189 (179–199)	193 (182–203)	448 (432–463)	458 (442–474)
20–24 years	44 (40–47)	44 (40–47)	175 (165–184)	177 (168–187)	412 (398–427)	419 (405–434)
25–29 years	40 (37–43)	40 (37–43)	160 (152–169)	162 (154–171)	378 (365–391)	383 (370–396)
30–34 years	36 (34–39)	36 (33–39)	146 (138–154)	147 (139–154)	345 (333–356)	347 (335–358)
35–39 years	33 (30–35)	32 (30–35)	132 (125–139)	132 (125–139)	313 (302–323)	312 (301–323)
40–44 years	29 (27–31)	29 (26–31)	118 (112–125)	118 (112–124)	280 (271–290)	277 (268–287)
45–49 years	25 (23–27)	25 (23–27)	105 (99–110)	104 (99–109)	248 (239–256)	242 (234–251)
50–54 years	22 (20–24)	22 (20–23)	91 (87–96)	90 (85–95)	215 (208–222)	208 (201–215)
55–59 years	18 (17–20)	18 (17–20)	78 (74–82)	77 (73–81)	184 (179–190)	177 (171–183)
60–64 years	15 (14–17)	15 (14–17)	66 (63–69)	65 (62–68)	157 (152–162)	149 (144–154)
65–69 years	13 (12–14)	13 (12–14)	55 (52–57)	54 (51–56)	131 (126–135)	124 (119–128)
70–74 years	10 (9–11)	10 (10–11)	45 (42–47)	44 (42–46)	106 (102–109)	101 (97–105)
75–79 years	8 (8–9)	9 (8–9)	35 (34–37)	35 (33–37)	84 (81–87)	81 (78–84)
80–84 years	7 (6–7)	7 (7–8)	29 (27–30)	29 (27–30)	67 (65–69)	66 (64–69)
85–89 years	6 (5–6)	6 (6–6)	24 (22–25)	24 (23–26)	54 (52–56)	55 (52–57)
90–94 years	5 (5–6)	5 (5–6)	20 (19–21)	22 (21–23)	45 (43–47)	48 (46–51)
95+ years	2 (2–2)	2 (2–2)	8 (8–9)	9 (8–9)	18 (17–19)	20 (18–21)

Note: 95% uncertainty ranges presented in parentheses accounting for age-weighting across countries within each income group

**Fig. 1:**
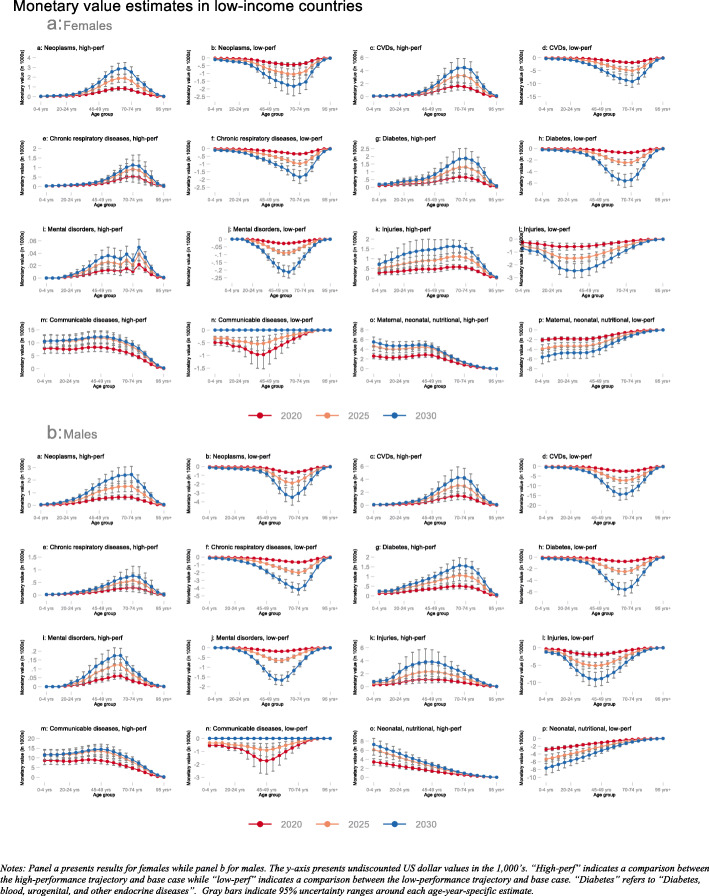
Monetary value (in 1000s USD) associated with switching from base-case to high-performance (“high-perf”) and low-performance (“low-perf”) trajectories; averaged across low-income countries in 2020, 2025, and 2030; disaggregated by age group and sex for eight disease categories. Notes: Panel **a** presents results for females while panel **b** for males. The y-axis presents undiscounted US dollar values in the 1000s. “High-perf” indicates a comparison between the high-performance trajectory and base-case while “low-perf” indicates a comparison between the low-performance trajectory and base-case. “Diabetes” refers to “Diabetes, blood, urogenital, and other endocrine diseases.” Gray bars indicate 95% uncertainty ranges around each age-year-specific estimate

**Fig. 2:**
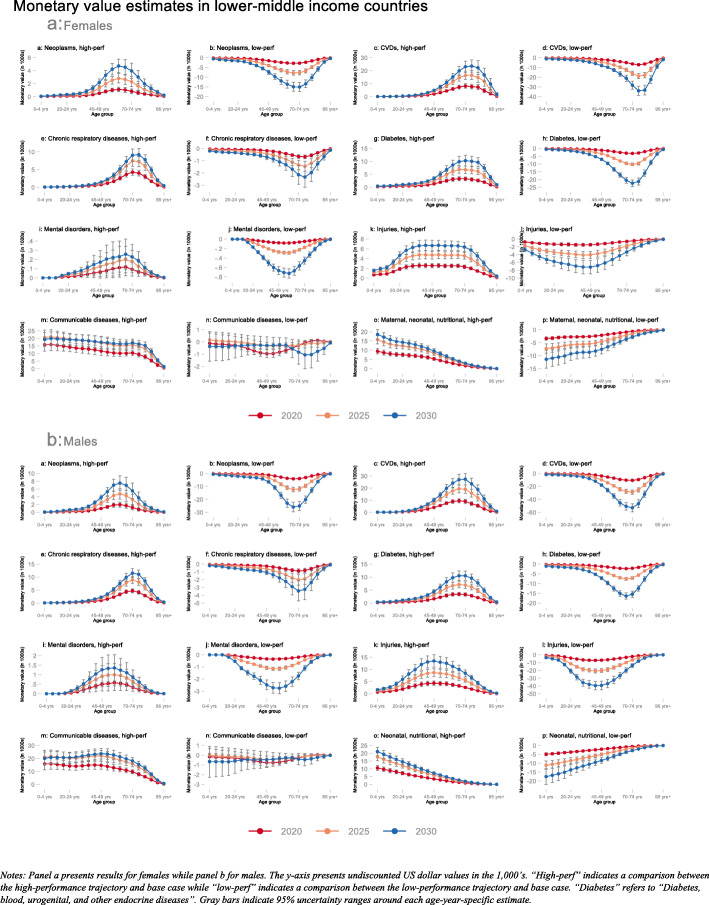
Monetary value (in 1000s USD) associated with switching from base-case to high-performance (“high-perf”) and low-performance (“low-perf”) trajectories; averaged across lower-middle-income countries in 2020, 2025, and 2030; disaggregated by age group and sex for eight disease categories. Notes: Panel **a** presents results for females while panel **b** for males. The y-axis presents undiscounted US dollar values in the 1000s. “High-perf” indicates a comparison between the high-performance trajectory and base-case while “low-perf” indicates a comparison between the low-performance trajectory and base-case. “Diabetes” refers to “Diabetes, blood, urogenital, and other endocrine diseases.” Gray bars indicate 95% uncertainty ranges around each age-year-specific estimate

**Fig. 3:**
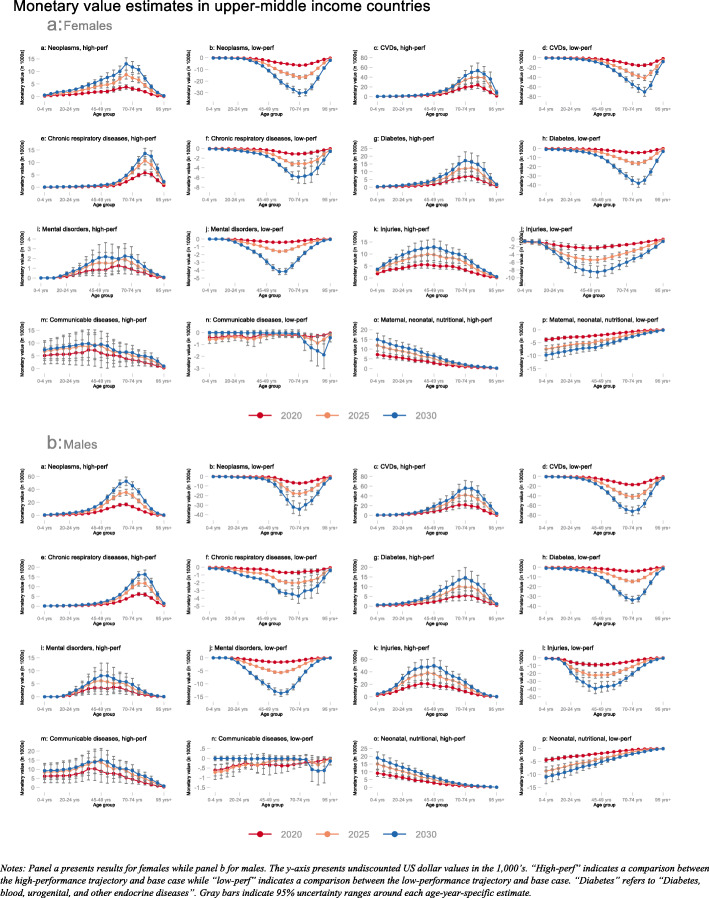
Monetary value (in 1000s USD) associated with switching from base-case to high-performance (“high-perf”) and low-performance (“low-perf”) trajectories; averaged across upper-middle-income countries in 2020, 2025, and 2030; disaggregated by age group and sex for eight disease categories. Notes: Panel **a** presents results for females while panel **b** for males. The y-axis presents undiscounted US dollar values in the 1000s. “High-perf” indicates a comparison between the high-performance trajectory and base case while “low-perf” indicates a comparison between the low-performance trajectory and base case. “Diabetes” refers to “Diabetes, blood, urogenital, and other endocrine diseases.” Gray bars indicate 95% uncertainty ranges around each age-year-specific estimate

Our primary substantive results are presented in Figs. 1, 2, and 3. All three figures illustrate undiscounted IEMV estimates for females (panel a) and males (panel b) in the years 2020 (red line), 2025 (orange line), and 2030 (blue line). All three figures provide IEMV estimates across all eight disease categories and for comparisons between the high-performance trajectory and the base-case (labeled “high-perf”) as well as the low-performance trajectory and the base-case (labeled “low-perf”).

Figure 1 shows that change in mortality between the base-case and low-performance trajectory is highest for CVDs among older females and males followed by other NCDs such as neoplasms and diabetes, blood, urogenital, and other endocrine diseases. For instance, in 2030, the increase in mortality risk in LICs as a result of being on the low-performance trajectory relative to the base-case among females aged 70–74 years had a IEMV of $9100 (95% UR $6800; $11,400) relative to $1700 ($1100; $2300) for neoplasms and $5400 ($4300; $6600) for diabetes, blood, urogenital, and other endocrine diseases. Among males, the change in mortality risk due to injury in LICs between the high-performance trajectory and the base-case was concentrated primarily in the adult age groups (30–60 years old) while the distribution seemed relatively more uniform for females. As expected, reducing the mortality risk due to CDs relative to the base-case was associated with high IEMV, especially among the youngest age groups. For example, among females aged 0–4 years, shifting the mortality rate from the base-case to the high-performance trajectory was valued at $7800 (95% UR $5900; $9800) in 2020, $10,600 ($8200; $13,000) in 2025, and $10,500 ($8300; $12,800) in 2030.

In LMICs, we observed a similar pattern of changes in mortality risk and their associated IEMVs (Fig. 2). Among NCDs, the change in mortality risk and, consequently, IEMV because of being on the high-performance trajectory relative to the base-case was highest for CVDs among both males and females, and especially in the older age groups. For instance, the IEMV for 70–74-year-old females in LMICs for being on the low-performance CVD mortality trajectory relative to the base-case in 2020 was $6400 (95% UR $5500; $7200), $16,100 ($13,900; $18,300) in 2025, and $28,300 ($24,200; $32,400) in 2030. IEMV estimates for either the low- or high-performance trajectory for CVDs were higher, in absolute terms, in comparison to being on the low- or high-performance trajectory for all other NCDs. Our results also suggest substantial mortality reductions due to CDs and, subsequently, IEMVs in the younger age groups for males and females because of being on the high-performance trajectory relative to the base-case.

In UMICs, our estimates for the absolute value of the IEMV were highest for CVDs among the older age groups when comparing the high/low-performance mortality trajectory against the base-case (Fig. 3). For instance, when comparing the low-performance trajectory with the base-case, IEMV estimates for CVD mortality changes among 70–74-year-old females were $6600 (95% UR $6000; $7100) in 2020, $16,800 ($15,300; $18,200) in 2025, and $30,300 ($27,200; $33,300) in 2030. Our IEMV estimates for increases in mortality risk because of being on the low-performance trajectory for diabetes, blood, urogenital, and other endocrine diseases were especially high as well. Reducing mortality due to injuries by being on the high-performance trajectory, especially among middle-aged males, led to IEMV estimates that were comparable to mortality reductions from being on the high-performance trajectory for CVDs.

Results from our sensitivity analyses are presented in Additional file 1: Web Appendix Figures A1-9. The distribution of IEMVs across the age-sex-year-country income groups is similar to our main results, although the magnitude of the estimates varies substantially depending on the assumptions made in estimating the country-year-specific VSLs. Specifically, for all contrasts between the high/low-performance trajectory and the base-case, the magnitude of the IEMV estimates across all age-sex groups were highest under the second sensitivity scenario where we set the VSL income elasticity parameter to 1 and the GNI per capita ratio to 160.

## Discussion

In this study, we presented an approach for estimating the monetary value associated with changes in disease-specific mortality risk across LICs, LMICs, and UMICs. Our methods built on the seminal work of Jamison and colleagues and incorporated best-practice recommendations from the published VSL literature [15, 19, 22]. We also presented an application of our methods using publicly available data from LICs, LMICs, and UMICs.

Two key results emerge from our analysis. First, from a substantive standpoint, our results suggest that the value of curbing both NCD- and CD-specific mortality may be very high for low- and middle-income countries, although the value of changes in mortality risk varies substantially by age group. For NCDs, curbing mortality due to CVDs among the older age groups may be particularly valuable in all countries; for CDs, curbing mortality among the younger age groups may be most important. Conversely, our results also suggest that countries could experience major setbacks if NCD control is not scaled up or if they lose sight of addressing the unfinished agenda of tackling CDs. This age-sex-specific disaggregation of IEMV is a strength of our approach because it can help policymakers identify groups that stand to benefit the most from focused interventions against specific diseases.

It is difficult to directly compare the magnitude of our estimated IEMVs with the published literature, primarily because the modeling approach we present is relatively novel. In a similar analysis, Bloom and colleagues also used VSL methods but provided aggregate level estimates which suggest that, by 2030, the economic burden of NCDs may be approximately $0.5 trillion in LICs, $5.3 trillion in LMICs, and $17.4 trillion in UMICs [25]. In a separate paper, Bloom and colleagues used macroeconomic modeling approaches to suggest that the economic burden of NCDs between 2010 and 2030 in China, Japan, and South Korea would be $16.0 trillion, $5.7 trillion, and $1.5 trillion, respectively [26]. In both cases, the estimation methods differ from ours in several ways. For example, the VSL methods used by Bloom and colleagues rely on a two-step process of estimating country-specific VSL. Specifically, they first regress VSL estimates from 12 countries reported in the literature on gross domestic product per capita and life expectancy at birth and then use this model to estimate VSL values in all other countries in their analysis [25]. In contrast, we estimate country-year-specific VSL values by scaling US VSL estimates as described in Eq. 1. Despite important methodological differences, what is clear from all these monetary assessments, including ours, is that NCDs and CDs will both exert a tremendous economic burden on LICs, LMICs, and UMICs unless mitigation measures, possibly targeted by age and sex, are put in place.

Although it is difficult to directly compare our substantive results with the published literature, what we can compare more directly are our VSL estimates. Viscusi and Masterman estimate average VSL in LICs, LMICs, and UMICs to be $107,000, $420,000, and $1.2 million, respectively [27]. In our study, the average VSL estimates over the study period from the primary and three sensitivity analyses included the figures presented by Viscusi and Masterman. For instance, in LICs, our primary VSL estimate was $321,300 while the estimates we used in our sensitivity analyses ranged from $64,100 to $201,700 (Additional file 1: Web Appendix Table A5). Such differences in the estimated VSL between the primary and sensitivity analyses explain the large range in our IEMV estimates. This leads to the second key insight from our analysis, which is that until empirical, country-specific VSL estimates are available, analyses that use our methods for estimating IEMVs, especially with the goal of subsequently engaging in priority setting and ROI analyses, should estimate VSLs using our primary methods as well as using all three sensitivity analyses.

There are four major limitations to the IEMV estimation methods we propose. First, our approach relies on extrapolating mortality rates but not underlying epidemiological and economic risk factors. The latter extrapolation approaches may potentially be more accurate, although even more modeling assumptions would be needed and data on epidemiological and economic risk factors may be scant in many low- and middle-income countries. Second, the high/low-performance mortality trajectories we propose reflect close to the best- and worst-case scenarios for changes in disease-specific mortality in LICs, LMICs, and UMICs. This is because they are respectively defined based on the 90th and 10th percentiles for mortality rates among countries in the next highest income group. Such scenarios may thus be unrealistic but nonetheless can help us understand the age-sex-year-country-specific IEMV bounds. Third, as highlighted by Robinson and colleagues, our valuation of mortality risk reductions may not reflect true population values for low- and middle-income countries as they are based on adapting US-grounded values [19, 22]. Fourth, we made a critical simplifying assumption that the mortality trajectory of one disease is independent of another. Future work would want to consider building in dynamic interactions between the various mortality trajectories across disease categories.

Our substantive results are also subject to a few limitations. First, the inputs underlying the computation of our mortality projections drew from modeled estimates and, as such, may not reflect the actual age-sex-disease-specific mortality rates in the countries we studied. Alternative data sources, from the World Health Organization notably, could be used to generate results for comparison purposes in future studies [28]. Second, our analysis did not account for changes in disease-specific mortality rates because of the COVID-19 pandemic since we do not fully know yet how the pandemic will affect mortality patterns across diseases and countries. Third, we used broad disease categories in our analysis, which may mask important heterogeneities in IEMV estimates by disease type. For example, among diseases classified under “Diabetes, blood, urogenital, and other endocrine diseases,” the primary contributors to mortality and IEMV are diabetes and chronic kidney diseases but not hemoglobinopathies and hemolytic anemias. Future research implementing our analytic framework should consider using more granularly defined disease categories to bring to light the heterogenous IEMV estimates associated with various morbidities.

Despite these limitations, our methods for estimating IEMV can be useful for policymakers—at least relative to mortality and life expectancy estimates solely—for several reasons: first, they can provide critical input into estimating the value for money and ROIs of various existing and proposed initiatives to curb NCDs and CDs; second, they can be combined with monetary estimates associated with other welfare outcomes and investments in other sectors (beyond the health sector only); and third, they can serve as an input into the calculations of a country’s full income, which may be particularly important for health policymakers in motivating the importance of investing in specific policies [12–14]. In addition, the approach we present in this paper is flexible in that it can be replicated at the regional, national, and subnational levels, which may be particularly helpful for priority setting. Finally, as shown by our substantive results, a key strength of our methodology is that it provides age-sex-disaggregated IEMVs, which may be helpful information for policymakers in designing health policies and programs, possibly independent of use into ROI studies or benefit-cost analyses.

## Conclusions

In this study, we presented a framework for systematically monetizing disease-specific mortality, which can then be used for conducting ROI studies, benefit-cost analyses, and various other priority setting exercises. This economic burden of disease framework is particularly salient today, with COVID-19 having a devastating impact on individuals affected by NCDs [29]. As analysts and policymakers attempt to estimate the full costs and benefits of COVID-19 and exercise fair priority setting, our framework may help point to high value for money interventions for optimized disease control [30–33].

## Supplementary Information

**Additional file 1.** Includes detailed methods and supplementary results.

## Data Availability

All data are publicly available from the Internet on the websites of the Global Burden of Disease study (IHME), the World Bank, and the United Nations Population Division. All data sources have been cited in the article.

## Abbreviations

CD: Communicable diseases
GBD: Global Burden of Disease
GNI: Gross national income
IEMV: Income-equivalent monetary value
LIC: Low-income countries
LMIC: Lower-middle-income countries
NCD: Non-communicable diseases
ROI: Return on investment
SDG: Sustainable Development Goals
SMU: Standardized mortality unit
UMIC: Upper-middle-income countries
UR: Uncertainty range
VSL: Value of a statistical life
VSMU: Value of standardized mortality units

## Footnotes

1 Thus, for LICs, the target mortality rate for 2030 under either the high- or low-performance trajectory was based on mortality rates from LMICs; for LMICs, it was based on mortality rates from UMICs; and for UMICs, it was based on mortality rates from high-income countries.

2 For example, in each country, we assumed that the mortality rate for neoplasms for females aged 15–19 years between 2017 and 2030 under the high/low-performance trajectory was not affected by the mortality rate for CVDs for the same age-sex group over the same time period.

3 For example, for a given LIC, we used the estimated mean growth rate among LICs in years prior to and including 2017 to estimate the GNI per capita for each country in each year between 2018 and 2030.
