# Supplementary material for: The economic value of changing mortality risk in low- and middle-income countries: a systematic breakdown by cause of death
Source: BMC Med. 2021 Jul 16;19:156. doi: 10.1186/s12916-021-02029-x (PMC8282406; doi:10.1186/s12916-021-02029-x)
Supplement: Supplementary file 1 — Additional file 1. Includes detailed methods and supplementary results. [file 12916_2021_2029_MOESM1_ESM.docx]

# **Web Appendix Detailed Methods**

As stated in the main text, we estimated monetary values associated with changes in mortality risk using three steps. For our first step, we estimated an age-sex-country-specific high- and low-performing mortality rate trajectory between 2017 and 2030; and we converted these rates into risks of death, and compared the risks of death in both these trajectories against a reference trajectory (“base-case). Second, we used the best-practice recommendations from the value of a statistical life (VSL) published literature to estimate country-year-specific VSL values, which we then used to calculate the value of changes in mortality risk between the high/low-performance trajectory and the reference trajectory. Third, we conducted multiple sensitivity analyses to test the robustness of our primary estimates to different assumptions we make when estimating country-year-specific VSL values.

We called our resulting monetary estimates “income-equivalent monetary value” (or IEMV). We calculate these values for low-income countries (LICs), lower-middle-income countries (LMICs), and upper-middle-income countries (UMICs). We calculated IEMVs for eight broad disease categories, which were defined using the Global Burden of Disease (GBD) study classification (Web Appendix Table A1). In what follows, we reproduce the main steps highlighted in the seminal work of Jamison and colleagues and follow best-practice recommendations from the VSL literature [1–5].

## ***Step 1:*** *Estimating mortality risk changes between high/low-performance trajectories and the base-case trajectory*

We defined mortality rate as the number of deaths due to disease $d$ divided by the population in age interval $[a; a+n$) in country $c$ at year $y$ for sex $s$ (females and males) separately. For all ages between 0 and 95, we have $n=5$ years; our final age group was an open age interval for individuals aged 95 years and older. We defined three mortality trajectories: a base-case which served as the reference; a high-performance trajectory; and a low-performance trajectory. The base-case referred to the annual age-sex-disease-specific mortality rate forecasts from the GBD 2016 study between 2017 and 2030 [6]. The high-performance trajectory was defined as achieving, by 2030, the age-sex-disease-specific mortality rates that corresponded to the 90^th^ percentile of the lowest mortality rates of countries in the next higher income grouping (for LICs, LMICs; for LMICs, UMICs; and for UMICs, high-income countries). Similarly, the low-performance trajectory was defined as achieving, by 2030, the rates that corresponded to the 10^th^ percentile of the lowest mortality rates of countries in the next higher income grouping (for LICs, LMICs; for LMICs, UMICs; and for UMICs, high-income countries).

We defined the high/low-performance trajectory for all age-sex groups for all diseases and across all countries in our data. To construct these trajectories, we identified the age-sex-disease-specific targets under the high/low-performance trajectory for each country based on our definition for these trajectories. Then, we log transformed (natural logarithm) the mortality rate in 2016 and the respective target mortality rate in 2030. Finally, we linearly interpolated the mortality rate for each year between 2017 and 2029. In constructing these disease-specific trajectories, we assumed that the trajectory for any given age-sex-disease-group was independent of the mortality rates of the other diseases.

For each of the three trajectories, we estimated overall age-sex-year-country-specific mortality rates ($m_{a,s,y,c}$) by assuming that

$m_{a,s,y,c}=\sum_{d} m_{a,s,d,y,c}$, [Eq. A1]

where $m_{a,s,d,y,c}$ refers to age-sex-disease-year-country-specific mortality rates. For the high- and low-performance scenarios, we computed Eq.A1 separately for each age-sex-disease-year-country category by assuming that for any given disease, mortality rates due to all other diseases remained the same as under the base-case.

We transformed age-sex-year-country-specific mortality rates into probabilities of dying ($q_{a,s,y,c}$) in age group $[a;a+n)$ for sex $s$ conditional on surviving until age $a$ as:

$$q_{a,s,y,c}=1-e^{\sum_{x=0}^{a} -n*m_{x,s,y,c}} , [Eq. A2]$$

where $m_{x,s,y,c}$ are age-sex-year-country-specific mortality rates for age group $x$ and $n$ is the age group size (e.g., 5 years). For the final, open age interval, we assume that $n=2.5$ years, which represents that, on average, individuals in this age group will live for 2.5 years.

Finally, we estimated the change in age-sex-year-country-specific mortality risk between the base-case and alternate trajectory $\left( \delta\left( q_{a,s,y,c}^{base};q_{a,s,y,c}^{alt} \right) \right)$ as:

$$\delta\left( q_{a,s,y,c}^{base},q_{a,s,y,c}^{alt} \right)=\left( e^{-n*m_{a,s,y,c}^{alt}}-e^{-n*m_{a,s,y,c}^{base}} \right)*{10}^{4} . [Eq. A3]$$

Scaling the change in mortality risk by ${10}^{4}$ in Eq. A3 allowed us to express the difference in mortality risk in terms of standardized mortality units (SMU). One SMU is defined as the change in mortality risk of ${10}^{-4}$ (or 1 per 10,000).

## ***Step 2:*** *Estimating the IEMV of changes in mortality risk*

In step 2, our goal was to estimate the monetary value associated with each age-sex-year-country-specific SMU and apply this value to the quantity by which mortality risk changed between the high/low-performance trajectory and the base-case. To do so, we used Jamison and colleagues as a starting point and estimated the monetary value associated with changes in mortality risk as [2]:

${MV}_{s,y,c}\sim\sum_{a=0}^{A} w_{a,s,y,c}*VSMU\left( a \right)*\delta\left( q_{a,s,y,c}^{base};q_{a,s,y,c}^{alt} \right)*n$, [Eq. A4]

where ${MV}_{s,y,c}$ is the monetary value associated with changes in mortality risk for individuals of sex $s$ in country $c$ and year $y$; $w_{a,s,y,c}$ is the density of age group $a$ of sex $s$; and $VSMU\left( a \right)$ is the value of a standardized mortality unit. We used population estimates from the United Nations Population Division’s World Population Prospects (WPP) as inputs for $w_{a,s,y,c} ADDIN ZOTERO\_ITEM CSL\_CITATION \{"citationID":"fpF5U0Ql","properties":\{"formattedCitation":"(7)","plainCitation":"(7)","noteIndex":0\},"citationItems":[\{"id":329,"uris":["http://zotero.org/users/local/331E7D2S/items/BFHVY2QI"],"uri":["http://zotero.org/users/local/331E7D2S/items/BFHVY2QI"],"itemData":\{"id":329,"type":"webpage","title":"World Population Prospects 2019","URL":"https://population.un.org/wpp/Download/Standard/Population/","author":[\{"literal":"United Nations Population Division"\}]\}\}],"schema":"https://github.com/citation-style-language/schema/raw/master/csl-citation.json"\} [7]$.

To estimate $VSMU\left( a \right)$, we proceeded in multiple steps. First, we computed the value of a statistical life ($VSL_{c,y}$) at age-group $[35, 39)$ in country $c$ and year $y$ as:

$$VSL_{c,y}=VSL_{base}*\left( \frac{I_{y,c}}{I_{y,base}} \right)^{\varepsilon} , [Eq. A5]$$

where $VSL_{base}$ is the average VSL in the US (in 2015), $I_{y,c}$ is the GNI per capita (adjusted for purchasing power parity, 2011 USD) in country $c$ and year $y$, $I_{y,base}$ is GNI per capita in year $y$ in the US (i.e. the reference country), and $\varepsilon$ refers to the VSL income elasticity. We used age-group $[35,39)$ as the reference age-group following Jamison et al. [2]. We set $VSL_{base}$ = $9.6 million and $\varepsilon$ = 1, and used estimates from the World Development Indicators (WDI) for the ratio of per capita GNIs [8,9].

Since WDI estimates for per capita GNI were only available through 2017, we derived a GNI value for each year between 2018-2030 using a three-step process. First, we computed the exponential growth rate in GNI per capita for each country in our sample using data from 2017 and before. Second, we estimated the mean GNI per capita growth rate by country income group using our values of the estimated exponential growth rate. Finally, we used the exponential growth rate formula and country income group-specific mean growth rate to estimate the yearly GNI per capita for each country between 2018 and 2030. All GNI per capita estimates were expressed in 2015 USD and were undiscounted.

We converted our country-year-specific estimates of $VSL_{c,y}$ into a VSMU estimate at age 35 as:

$$VSMU_{c,y}\left( 35 \right)=VSL_{c,y}*{10}^{-4} , [Eq. A6]$$

and, we derived VSMUs at different ages $a$ as:

$$VSMU_{c,y}\left( a \right)=\frac{e_{a,s,y,c}}{e_{35,s,y,c}}VSMU_{c,y}\left( 35 \right) . [Eq. A7]$$

$e_{a,s,y,c}$ refers to the age-sex-year-country-specific life expectancy. We estimated life expectancy using base-case mortality rates and standard life table methods for estimating period life expectancy.

## *Step 3: Sensitivity analyses*

We closely followed the recommendations put forward by Robinson and colleagues in estimating VSL under three additional scenarios [3]. First, using $\varepsilon=1.5$, $VSL_{base}$= $9.4 million, and a constant US GNI per capita estimate ($57,900). If this yielded values < 20 times GNI per capita of a given country, we used 20 times GNI per capita as the VSL. Second, we set country-specific VSL as 160 times GNI per capita where 160 is the ratio of VSL to GNI per capita in the US. Third, we set country-specific VSL as 100 times GNI per capita where 100 is the ratio of VSL to GNI per capita among countries from the Organization for Economic Cooperation and Development (OECD). In both second and third scenarios, we set $\varepsilon$ to 1.

**References**

1. Jamison DT, Summers LH, Alleyne G, Arrow KJ, Berkley S, Binagwaho A, et al. Global health 2035: a world converging within a generation. The Lancet. 2013 Dec;382(9908):1898–955.

2. Jamison DT, Summers LH, Alleyne G, Arrow KJ, Berkley S, Binagwaho A, et al. Supplementary Appendix 3 to Global health 2035: a world converging within a generation. Lancet. 2013 Dec 7;382(9908):1898–955. Available from: https://www.thelancet.com/cms/10.1016/S0140-6736(13)62105-4/attachment/b94f0afe-5ebe-4210-8ff7-c404e6a5bd64/mmc3.pdf

3. Robinson LA, Hammitt JK, O’Keeffe L. Valuing Mortality Risk Reductions in Global Benefit-Cost Analysis. Journal of Benefit-Cost Analysis. 2019 Jan 15;10(S1):15–50.

4. Robinson LA, Hammitt JK, Cecchini M, Chalkidou K, Claxton K, Cropper M, et al. Reference Case Guidelines for Benefit-Cost Analysis in Global Health and Development [Internet]. Harvard T. H. Chan School of Public Health; 2019 May. Available from: https://cdn1.sph.harvard.edu/wp-content/uploads/sites/2447/2019/05/BCA-Guidelines-May-2019.pdf

5. Robinson LA, Hammitt JK, Jamison DT, Walker DG. Conducting Benefit-Cost Analysis in Low- and Middle-Income Countries: Introduction to the Special Issue. J Benefit Cost Anal. 2019;10(Suppl 1):1–14.

6. Institute for Health Metrics and Evaluation. GBD Foresight | Viz Hub [Internet]. Available from: https://vizhub.healthdata.org/gbd-foresight/

7. United Nations Population Division. World Population Prospects 2019 [Internet]. Available from: https://population.un.org/wpp/Download/Standard/Population/

8. The World Bank. World Development Indicators: Data Catalog [Internet]. Available from: https://datacatalog.worldbank.org/dataset/world-development-indicators

9. Viscusi WK, Masterman CJ. Income Elasticities and Global Values of a Statistical Life. Journal of Benefit-Cost Analysis. 2017 ed;8(2):226–50.

# **Web Appendix: Countries excluded from our analysis**

Due to lack of data on GNI, we excluded from our analysis the following countries: the Democratic People’s Republic of Korea (low-income country or LIC), Somalia (LIC), Djibouti (lower-middle-income country or LMIC), Syria (LMIC), American Samoa (upper-middle-income country or UMIC), and Cuba (UMIC). We also excluded Dominica and the Marshall Islands (two UMICs) from our analysis due to lack of population data. Finally, Kosovo was excluded from our analysis because its mortality schedule was not available from the Global Burden of Disease study.

**Web Appendix Table A1. Disease categories included in the analysis and their corresponding Global Burden of Disease 2016 study code.**

| **Disease category** | **Diseases included in category** | **GBD (2016) code** |
| --- | --- | --- |
| Neoplasms | Lip and oral cavity cancer; Nasopharynx cancer; Other pharynx cancer; Esophageal cancer; Stomach cancer; Colon and rectum cancer; Liver cancer; Gallbladder and biliary tract cancer; Pancreatic cancer; Larynx cancer; Tracheal, bronchus, and lung cancer; Malignant skin melanoma; Non-melanoma skin cancer; Breast cancer; Cervical cancer; Uterine cancer; Ovarian cancer; Prostate cancer; Testicular cancer; Kidney cancer; Bladder cancer; Brain and central nervous system cancer; Thyroid cancer; Mesothelioma; Hodgkin lymphoma; Non-Hodgkin lymphoma; Multiple myeloma; Leukemia; Other neoplasms | B.1 |
| Cardiovascular diseases | Rheumatic heart disease; Ischemic heart disease; Stroke; Hypertensive heart disease; Cardiomyopathy and myocarditis; Atrial fibrillation and flutter; Aortic aneurysm; Peripheral artery disease; Endocarditis; Other cardiovascular and circulatory diseases | B.2 |
| Chronic respiratory diseases | Chronic obstructive pulmonary disease; Pneumoconiosis; Asthma; Interstitial lung disease and pulmonary sarcoidosis | B.3 |
| Diabetes, blood, urogenital, and other endocrine diseases | Diabetes mellitus; Acute glomerulonephritis; Chronic kidney disease; Urinary diseases and male infertility; Gynecological diseases; Hemoglobinopathies and hemolytic anemias; Endocrine, metabolic, blood, and immune disorders | B.8 |
| Mental disorders | Alcohol use disorders; Drug use disorders; Eating disorders | B.7 |
| Injuries | Transport injuries; Unintentional injuries; Self-harm and interpersonal violence; Forces of nature, conflict, and terrorism, and executions and police conflict | C.1, C.2, C.3, C.4 |
| Communicable diseases | HIV/AIDS and tuberculosis; Diarrhea, lower respiratory, and other common infectious diseases; Neglected tropical diseases and malaria; Other communicable, maternal, neonatal, and nutritional diseases | A.1, A.2, A.3, A.17 |
| Maternal, neonatal, and nutritional diseases | Maternal disorders; Neonatal disorders; Nutritional deficiencies | A.4, A.5, A.6 |

*Note: GBD = Global Burden of Disease. Disease code and categories are available on the GBD Foresight tool (URL: https://vizhub.healthdata.org/gbd-foresight/)*

**Web Appendix Table A2 Age-sex specific value of standardized mortality units (VSMU) in sensitivity analysis #1 averaged across the study period and stratified by World Bank country income groupings**

|  | Low-income country | | Lower-middle-income country | | Upper-middle-income country | |
| --- | --- | --- | --- | --- | --- | --- |
| Age group | **Females** | **Males** | **Females** | **Males** | **Females** | **Males** |
| <5 years | 12 (10-13) | 12 (11-14) | 95 (86-103) | 98 (89-106) | 345 (324-366) | 359 (337-381) |
| 5-9 years | 11 (10-13) | 11 (10-13) | 89 (81-97) | 92 (84-100) | 323 (304-343) | 334 (314-355) |
| 10-14 years | 10 (9-12) | 11 (9-12) | 84 (76-91) | 86 (78-93) | 301 (283-320) | 310 (291-328) |
| 15-19 years | 10 (9-11) | 10 (9-11) | 78 (71-85) | 79 (72-86) | 279 (262-296) | 285 (268-302) |
| 20-24 years | 9 (8-10) | 9 (8-10) | 72 (66-78) | 73 (67-79) | 257 (241-272) | 261 (245-276) |
| 25-29 years | 8 (7-9) | 8 (7-9) | 66 (60-72) | 67 (61-72) | 235 (221-249) | 238 (224-252) |
| 30-34 years | 8 (7-8) | 7 (7-8) | 60 (55-66) | 61 (56-66) | 214 (202-227) | 215 (203-228) |
| 35-39 years | 7 (6-8) | 7 (6-7) | 55 (50-59) | 55 (50-59) | 195 (183-206) | 194 (183-205) |
| 40-44 years | 6 (5-7) | 6 (5-7) | 49 (45-54) | 49 (45-53) | 175 (165-185) | 173 (163-183) |
| 45-49 years | 5 (5-6) | 5 (5-6) | 44 (40-48) | 43 (40-47) | 155 (145-164) | 151 (142-160) |
| 50-54 years | 5 (4-5) | 5 (4-5) | 38 (35-42) | 38 (35-41) | 134 (126-141) | 129 (122-137) |
| 55-59 years | 4 (3-4) | 4 (3-4) | 33 (30-36) | 32 (30-35) | 114 (108-121) | 109 (103-115) |
| 60-64 years | 3 (3-4) | 3 (3-4) | 28 (26-30) | 27 (25-30) | 97 (92-103) | 92 (87-98) |
| 65-69 years | 3 (2-3) | 3 (2-3) | 23 (21-25) | 23 (21-25) | 82 (77-86) | 77 (72-82) |
| 70-74 years | 2 (2-2) | 2 (2-2) | 19 (17-20) | 19 (17-20) | 66 (62-70) | 63 (59-67) |
| 75-79 years | 2 (2-2) | 2 (2-2) | 15 (14-16) | 15 (14-16) | 53 (50-56) | 50 (47-54) |
| 80-84 years | 1 (1-2) | 1 (1-2) | 12 (11-13) | 12 (11-13) | 42 (40-45) | 41 (39-44) |
| 85-89 years | 1 (1-1) | 1 (1-1) | 10 (9-11) | 10 (9-11) | 34 (32-36) | 34 (32-37) |
| 90-94 years | 1 (1-1) | 1 (1-1) | 9 (8-9) | 9 (8-10) | 29 (27-31) | 30 (28-33) |
| 95+ years | 0 (0-1) | 0 (0-1) | 3 (3-4) | 4 (3-4) | 11 (10-12) | 12 (11-13) |

*Note: 95% uncertainty ranges presented in parentheses accounting for age-weighting across countries within each income group. Sensitivity analysis #1 refers to an analysis conducted by setting* $\varepsilon=1.5$, $VSL_{base}$= $9.4 million, *and a constant US GNI per capita estimate ($57,900). If this yields values < 20 times GNI per capita of a given country, we use 20 times GNI per capita as the VSL.*

**Web Appendix Table A3 Age-sex specific value of standardized mortality units (VSMU) in sensitivity analysis #2 averaged across the study period and stratified by World Bank Country Income Groupings 2016**

|  | Low-income country | | Lower-middle-income country | | Upper-middle-income country | |
| --- | --- | --- | --- | --- | --- | --- |
| Age group | **Females** | **Males** | **Females** | **Males** | **Females** | **Males** |
| <5 years | 60 (55-65) | 60 (55-66) | 237 (224-251) | 245 (231-259) | 565 (543-587) | 589 (566-612) |
| 5-9 years | 56 (52-61) | 57 (52-61) | 223 (211-236) | 230 (216-243) | 530 (510-551) | 549 (528-570) |
| 10-14 years | 52 (48-57) | 53 (48-57) | 209 (197-221) | 214 (201-226) | 495 (475-514) | 509 (489-528) |
| 15-19 years | 49 (45-53) | 49 (45-53) | 194 (183-205) | 198 (187-209) | 458 (441-476) | 469 (451-487) |
| 20-24 years | 45 (41-48) | 45 (41-48) | 179 (169-189) | 182 (172-192) | 422 (406-438) | 430 (414-446) |
| 25-29 years | 41 (38-44) | 41 (38-44) | 164 (155-173) | 166 (157-175) | 387 (372-402) | 392 (377-407) |
| 30-34 years | 37 (34-40) | 37 (34-40) | 150 (142-158) | 151 (143-159) | 353 (340-366) | 355 (342-368) |
| 35-39 years | 33 (31-36) | 33 (30-36) | 136 (128-143) | 136 (128-143) | 320 (308-332) | 320 (308-331) |
| 40-44 years | 30 (27-32) | 29 (27-32) | 122 (115-128) | 121 (114-127) | 287 (277-298) | 284 (273-294) |
| 45-49 years | 26 (24-28) | 26 (24-28) | 108 (102-113) | 107 (101-112) | 254 (244-263) | 248 (239-258) |
| 50-54 years | 22 (21-24) | 22 (20-24) | 94 (89-99) | 92 (87-97) | 220 (212-228) | 214 (206-221) |
| 55-59 years | 19 (17-20) | 19 (17-20) | 80 (76-85) | 79 (75-83) | 189 (182-196) | 181 (175-188) |
| 60-64 years | 16 (14-17) | 16 (15-17) | 68 (64-71) | 67 (63-70) | 161 (155-166) | 153 (147-158) |
| 65-69 years | 13 (12-14) | 13 (12-14) | 56 (53-59) | 55 (52-58) | 134 (129-139) | 127 (122-132) |
| 70-74 years | 11 (10-11) | 11 (10-12) | 46 (43-48) | 45 (43-47) | 108 (104-112) | 103 (99-108) |
| 75-79 years | 9 (8-9) | 9 (8-9) | 36 (34-38) | 36 (34-38) | 86 (83-89) | 83 (80-87) |
| 80-84 years | 7 (6-8) | 7 (7-8) | 29 (28-31) | 30 (28-31) | 69 (66-71) | 68 (65-71) |
| 85-89 years | 6 (5-6) | 6 (6-7) | 24 (23-26) | 25 (24-26) | 55 (53-58) | 56 (54-59) |
| 90-94 years | 5 (5-6) | 6 (5-6) | 21 (20-22) | 22 (21-23) | 46 (44-49) | 50 (47-52) |
| 95+ years | 2 (2-2) | 2 (2-3) | 8 (8-9) | 9 (8-10) | 18 (17-19) | 20 (19-21) |

*Note: 95% uncertainty ranges presented in parentheses accounting for age-weighting across countries within each income group. Sensitivity analysis #2 refers to an analysis conducted by setting* $\varepsilon=1$ *and country-specific VSL as 160 times GNI per capita where 160 is the ratio of VSL to GNI per capita in the US.*

**Web Appendix Table A4 Age-sex specific value of standardized mortality units (VSMU) in sensitivity analysis #3 averaged across the study period and stratified by World Bank Country Income Groupings 2016**

|  | Low-income country | | Lower-middle-income country | | Upper-middle-income country | |
| --- | --- | --- | --- | --- | --- | --- |
| Age group | **Females** | **Males** | **Females** | **Males** | **Females** | **Males** |
| <5 years | 12 (9-15) | 12 (9-15) | 95 (86-103) | 98 (89-106) | 345 (331-358) | 359 (344-373) |
| 5-9 years | 11 (8-14) | 11 (8-14) | 89 (81-97) | 92 (84-100) | 323 (311-336) | 334 (321-348) |
| 10-14 years | 10 (8-13) | 11 (8-13) | 84 (76-91) | 86 (78-93) | 301 (290-313) | 310 (297-322) |
| 15-19 years | 10 (7-12) | 10 (7-12) | 78 (71-85) | 79 (72-86) | 279 (268-290) | 285 (274-296) |
| 20-24 years | 9 (7-11) | 9 (7-11) | 72 (66-78) | 73 (67-79) | 257 (247-267) | 261 (251-271) |
| 25-29 years | 8 (6-10) | 8 (6-10) | 66 (60-72) | 67 (61-73) | 235 (226-244) | 238 (229-247) |
| 30-34 years | 8 (6-9) | 7 (6-9) | 60 (55-66) | 61 (56-66) | 214 (206-222) | 215 (207-223) |
| 35-39 years | 7 (5-8) | 7 (5-8) | 55 (50-59) | 55 (50-59) | 195 (187-202) | 194 (187-201) |
| 40-44 years | 6 (5-8) | 6 (4-7) | 49 (45-54) | 49 (45-53) | 175 (168-181) | 173 (166-179) |
| 45-49 years | 5 (4-7) | 5 (4-7) | 44 (40-48) | 43 (40-47) | 155 (149-160) | 151 (145-157) |
| 50-54 years | 5 (3-6) | 5 (3-6) | 38 (35-42) | 38 (35-41) | 134 (129-139) | 129 (124-134) |
| 55-59 years | 4 (3-5) | 4 (3-5) | 33 (30-36) | 32 (30-35) | 114 (110-119) | 109 (105-113) |
| 60-64 years | 3 (2-4) | 3 (2-4) | 28 (26-30) | 27 (25-30) | 97 (94-101) | 92 (89-96) |
| 65-69 years | 3 (2-3) | 3 (2-3) | 23 (21-25) | 23 (21-25) | 82 (79-85) | 77 (74-80) |
| 70-74 years | 2 (2-3) | 2 (2-3) | 19 (17-20) | 19 (17-20) | 66 (64-69) | 63 (60-65) |
| 75-79 years | 2 (1-2) | 2 (1-2) | 15 (14-16) | 15 (14-16) | 53 (51-55) | 50 (48-53) |
| 80-84 years | 1 (1-2) | 1 (1-2) | 12 (11-13) | 12 (11-13) | 42 (41-44) | 41 (39-43) |
| 85-89 years | 1 (1-1) | 1 (1-2) | 10 (9-11) | 10 (9-11) | 34 (33-36) | 34 (33-36) |
| 90-94 years | 1 (1-1) | 1 (1-1) | 9 (8-9) | 9 (8-10) | 29 (28-30) | 30 (29-32) |
| 95+ years | 0 (0-1) | 0 (0-1) | 3 (3-4) | 4 (3-4) | 11 (11-12) | 12 (11-13) |

*Note: 95% uncertainty ranges presented in parentheses accounting for age-weighting across countries within each income group. Sensitivity analysis #3 refers to an analysis conducted by setting* $\varepsilon=1$ *and country-specific VSL as 100 times GNI per capita where 100 is the ratio of VSL to GNI per capita among countries from the Organization for Economic Cooperation and Development (OECD).*

**Web Appendix Table A5 Average value of statistical life estimate over the study period (2017-2030) by country income grouping for our primary analysis and three sensitivity analyses**

|  | **Primary VSL estimate** | **VSL estimate for sensitivity analysis #1** | **VSL estimate for sensitivity analysis #2** | **VSL estimate for sensitivity analysis #3** |
| --- | --- | --- | --- | --- |
| **LIC** | $321,300 | $64,100 | $322,800 | $201,700 |
| **LMIC** | $1,156,300 | $453,100 | $1,163,800 | $727,400 |
| **UMIC** | $2,769,800 | $1,623,100 | $2,784,100 | $1,740,100 |

*Note: VSL = value of statistical life. LIC = low-income country; LMIC = lower-middle-income country; UMIC = upper-middle-income country. All values reported in undiscounted, 2015 USD.*

**Web Appendix Figure A1 Monetary value (in 1000s USD) associated with switching from base-case to high-performance (“high-perf”) and low-performance (“low-perf”) trajectories; averaged across low-income countries in 2020, 2025, and 2030; disaggregated by age group and sex for eight disease categories [Sensitivity analysis #1]**

**

*Notes: Panel a presents results for females while panel b presents results for males. The y-axis presents undiscounted dollar values in 1,000’s. “High-perf” indicates a comparison between the high-performance trajectory and base case, while “low-perf” indicates a comparison between the low-performance trajectory and base case. “Diabetes” refers to “Diabetes, blood, urogenital, and other endocrine diseases”. Sensitivity analysis #1 refers to an analysis conducted by setting* $\varepsilon=1.5$, $VSL_{base}$= $9.4 million, *and a constant US GNI per capita estimate ($57,900). If this yields values < 20 times GNI per capita of a given country, we use 20 times GNI per capita as the VSL. Gray bars indicate 95% uncertainty ranges around each age-year-specific estimate.*

**Web Appendix Figure A2 Monetary value (in 1000s USD) associated with switching from base case to high-performance (“high-perf”) and low-performance (“low-perf”) trajectories respectively averaged across low-income countries in 2020, 2025, and 2030; disaggregated by age group and sex for all disease categories [Sensitivity analysis #2]**

*Notes: Panel a presents results for females while panel b for males. The y-axis presents undiscounted dollar values in the 1,000’s. “High-perf” indicates a comparison between the high-performance trajectory and base case while “low-perf” indicates a comparison between the low-performance trajectory and base case. “Diabetes” refers to “Diabetes, blood, urogenital, and other endocrine diseases”. Sensitivity analysis #2 refers to an analysis conducted by setting* $\varepsilon=1$ *and country-specific VSL as 160 times GNI per capita where 160 is the ratio of VSL to GNI per capita in the US. Gray bars indicate 95% uncertainty ranges around each age-year-specific estimate.*

**Web Appendix Figure A3 Monetary value (in 1000s USD) associated with switching from base case to high-performance (“high=-perf”) and low-performance (“low-perf”) trajectories respectively averaged across low-income countries in 2020, 2025, and 2030; disaggregated by age-group and sex for all disease categories [Sensitivity analysis #3]**

**

*Notes: Panel a presents results for females while panel b for males. The y-axis presents undiscounted dollar values in the 1,000’s. “High-perf” indicates a comparison between the high-performance trajectory and base case while “low-perf” indicates a comparison between the low-performance trajectory and base case. “Diabetes” refers to “Diabetes, blood, urogenital, and other endocrine diseases”. Sensitivity analysis #3 refers to an analysis conducted by setting* $\varepsilon=1$ *and country-specific VSL as 100 times GNI per capita where 100 is the ratio of VSL to GNI per capita among countries from the Organization for Economic Cooperation and Development (OECD). Gray bars indicate 95% uncertainty ranges around each age-year-specific estimate.*

**Web Appendix Figure A4 Monetary value (in 1000s USD) associated with switching from base case to high-performance (‘high-perf”) and low-performance (“low-perf”) trajectories respectively averaged across lower-middle income countries in 2020, 2025, and 2030; disaggregated by age-group and sex for all disease categories [Sensitivity analysis #1]**

*Notes: Panel a presents results for females while panel b for males. The y-axis presents undiscounted dollar values in the 1,000’s. “High-perf” indicates a comparison between the high-performance trajectory and base case while “low-perf” indicates a comparison between the low-performance trajectory and base case. “Diabetes” refers to “Diabetes, blood, urogenital, and other endocrine diseases”. Sensitivity analysis #1 refers to an analysis conducted by setting* $\varepsilon=1.5$, $VSL_{base}$= $9.4 million, *and a constant US GNI per capita estimate ($57,900). If this yields values < 20 times GNI per capita of a given country, we use 20 times GNI per capita as the VSL. Gray bars indicate 95% uncertainty ranges around each age-year-specific estimate.*

**Web Appendix Figure A5 Monetary value (in 1000s USD) associated with switching from base case to high-performance (‘high-perf”) and low-performance (“low-perf”) trajectories respectively averaged across lower-middle income countries in 2020, 2025, and 2030; disaggregated by age-group and sex for all disease categories [Sensitivity analysis #2]**

*Notes: Panel a presents results for females while panel b for males. The y-axis presents undiscounted dollar values in the 1,000’s. “High-perf” indicates a comparison between the high-performance trajectory and base case while “low-perf” indicates a comparison between the low-performance trajectory and base case. “Diabetes” refers to “Diabetes, blood, urogenital, and other endocrine diseases”. Sensitivity analysis #2 refers to an analysis conducted by setting* $\varepsilon=1$ *and country-specific VSL as 160 times GNI per capita where 160 is the ratio of VSL to GNI per capita in the US. Gray bars indicate 95% uncertainty ranges around each age-year-specific estimate.*

**Web Appendix Figure A6 Monetary value (in 1000s USD) associated with switching from base case to high-performance (‘high-perf”) and low-performance (“low-perf”) trajectories respectively averaged across lower-middle income countries in 2020, 2025, and 2030; disaggregated by age-group and sex for all disease categories [Sensitivity analysis #3]**

**

*Notes: Panel a presents results for females while panel b for males. The y-axis presents undiscounted dollar values in the 1,000’s. “High-perf” indicates a comparison between the high-performance trajectory and base case while “low-perf” indicates a comparison between the low-performance trajectory and base case. “Diabetes” refers to “Diabetes, blood, urogenital, and other endocrine diseases”. Sensitivity analysis #3 refers to an analysis conducted by setting* $\varepsilon=1$ *and country-specific VSL as 100 times GNI per capita where 100 is the ratio of VSL to GNI per capita among countries from the Organization for Economic Cooperation and Development (OECD). Gray bars indicate 95% uncertainty ranges around each age-year-specific estimate.*

**Web Appendix Figure A7 Monetary value (in 1000s USD) associated with switching from base case to high-performance (‘high-perf”) and low-performance (“low-perf”) trajectories respectively averaged across upper-middle income countries in 2020, 2025, and 2030; disaggregated by age-group and sex for all disease categories [Sensitivity analysis #1]**

**

*Notes: Panel a presents results for females while panel b for males. The y-axis presents undiscounted dollar values in the 1,000’s. “High-perf” indicates a comparison between the high-performance trajectory and base case while “low-perf” indicates a comparison between the low-performance trajectory and base case. “Diabetes” refers to “Diabetes, blood, urogenital, and other endocrine diseases”. Sensitivity analysis #1 refers to an analysis conducted by setting* $\varepsilon=1.5$, $VSL_{base}$= $9.4 million, *and a constant US GNI per capita estimate ($57,900). If this yields values < 20 times GNI per capita of a given country, we use 20 times GNI per capita as the VSL. Gray bars indicate 95% uncertainty ranges around each age-year-specific estimate.*

**Web Appendix Figure A8 Monetary value (in 1000s USD) associated with switching from base case to high-performance (‘high-perf”) and low-performance (“low-perf”) trajectories respectively averaged across upper-middle income countries in 2020, 2025, and 2030; disaggregated by age-group and sex for all disease categories [Sensitivity analysis #2]**

*Notes: Panel a presents results for females while panel b for males. The y-axis presents undiscounted dollar values in the 1,000’s. “High-perf” indicates a comparison between the high-performance trajectory and base case while “low-perf” indicates a comparison between the low-performance trajectory and base case. “Diabetes” refers to “Diabetes, blood, urogenital, and other endocrine diseases”. Sensitivity analysis #2 refers to an analysis conducted by setting* $\varepsilon=1$ *and country-specific VSL as 160 times GNI per capita where 160 is the ratio of VSL to GNI per capita in the US. Gray bars indicate 95% uncertainty ranges around each age-year-specific estimate.*

**Web Appendix Figure A9 Monetary value (in 1000s USD) associated with switching from base case to high-performance (‘high-perf”) and low-performance (“low-perf”) trajectories respectively averaged across upper-middle income countries in 2020, 2025, and 2030; disaggregated by age-group and sex for all disease categories [Sensitivity analysis #3]**

*Notes: Panel a presents results for females while panel b for males. The y-axis presents undiscounted dollar values in the 1,000’s. “High-perf” indicates a comparison between the high-performance trajectory and base case while “low-perf” indicates a comparison between the low-performance trajectory and base case. “Diabetes” refers to “Diabetes, blood, urogenital, and other endocrine diseases”. Sensitivity analysis #3 refers to an analysis conducted by setting* $\varepsilon=1$ *and country-specific VSL as 100 times GNI per capita where 100 is the ratio of VSL to GNI per capita among countries from the Organization for Economic Cooperation and Development (OECD). Gray bars indicate 95% uncertainty ranges around each age-year-specific estimate.*
